# Morphological Variation and Spatial Metabolic Variations in *Rhodiola sachalinensis* A.Bor. in Different Natural Distribution Areas

**DOI:** 10.3390/plants13040467

**Published:** 2024-02-06

**Authors:** Qiuyang Chang, Xu Liu, Yi Li, Wen Zhao, Zhonghua Tang, Yang Liu, Liqiang Mu

**Affiliations:** 1 School of Forestry, Northeast Forestry University, Harbin 150040, Chinaliuxu19981209@nefu.edu.cn (X.L.);; 2 College of Chemistry, Chemical Engineering and Resource Utilization, Northeast Forestry University, Harbin 150040, China; tangzh@nefu.edu.cn; 3 Engineering Research Center of Agricultural Microbiology Technology, Ministry of Education, Heilongjiang University, Harbin 150500, China

**Keywords:** morphological variation, metabolic characteristics, *Rhodiola sachalinensis* A.Boriss., GS-MS

## Abstract

To explore the genetic diversity and metabolic characteristics among different locations of wild *Rhodiola sachalinensis* A.Boriss., we collected specimens from two sites (DHL: 128°23′06″ N, 44°26′31″ E; FHS: 127°59′26″ N, 44°7′22″ E) and measured various biological traits, such as leaf length, leaf width, and plant height. We conducted metabolic analyses to investigate variations among different plant parts. Our study revealed that while the various plant parts of wild *R. sachalinensis* A.Boriss. from these two locations showed overall numerical similarities, they exhibited relatively high coefficients of variation in traits such as leaf length, leaf width, plant height, and stem thickness. Furthermore, utilizing gas chromatography–mass spectrometry (GS-MS), we detected significant differences in primary metabolites among different plant parts from both locations. Using orthogonal partial least squares discriminant analysis (OPLS-DA), we identified 42 and 34 different metabolites in the roots, stems, and leaves of plants from the DHL site and 62 and 50 different metabolites in the roots, stems, and leaves of plants from the FHS site. Metabolic heatmaps suggested that sugar metabolism was more active in the roots compared to other plant parts. Through KEGG pathway analysis, we determined that the primary metabolic differences were concentrated in the citric acid cycle (TCA cycle) and amino acid metabolism, including pathways related to glycine, serine, and threonine metabolism, as well as alanine, aspartate, and glutamate metabolism. These findings indicate that wild *R. sachalinensis* A.Boriss. plants from different locations not only exhibit significant variations in biological traits but also demonstrate notable distinctions in the distribution of primary metabolites among different plant parts.

## 1. Introduction

*Rhodiola sachalinensis* A.Boriss., a perennial herb belonging to the Crassulaceae family in the Rhodiola genus, is a widely used medicinal plant [1]. It features robust roots, erect stems, and membranous leaves and grows to a height of 6–30 cm on flowering stems. This plant exhibits dioecious reproductive structures, with a flowering period from April to June and fruiting occurring from July to September. *R. sachalinensis*, also known as “Kuril Red Ginseng,” is primarily found in North America, Europe, and the northwestern regions of Asia [2]. In China, it is mainly distributed in Jilin and Heilongjiang provinces north of the Changbai Mountains.

*R. sachalinensis* is famous as the “Eastern God Herb” for its ability to ameliorate high-altitude sickness caused by hypoxia, alleviate depression, and improve sleep [3,4]. Extensive research in the fields of healthcare and neurology has also explored its various other functions, such as antioxidant properties, enhanced adaptability, and improved memory [5,6,7,8]. Active compounds found in *R. sachalinensis*, including salidroside and tyrosol, are of paramount importance. However, the significant variations in their content among different plant parts underscore the importance of genetic and compositional studies across various plant components.

The study of plant genetic diversity employs a range of methods from macro to micro levels, with morphological research often being the most direct and effective approach [9]. Many scholars have conducted genetic diversity analyses based on plant phenotypes. For example, Kjær Anders (2004) conducted research on the phenotypic morphology and genetic relationships of sago palm (Metroxylon sagu Rottb.) [10], and Y.P. Du (2014) conducted surveys and evaluations of lily resources [11]. Through the analysis of morphological data, we can gain insight into the genetic patterns of plants in the most intuitive way, which is highly advantageous for understanding the genetic characteristics and relationships of the studied plant.

Metabolomics primarily focuses on comprehensive studies of endogenous low-molecular-weight metabolites, providing a method for detecting overall changes in metabolite profiles [12,13]. Metabolomics can also unveil metabolic differences between different plant parts and their interconnections [14]. Major analytical platforms in metabolomics research include gas chromatography–mass spectrometry (GC-MS), liquid chromatography–mass spectrometry (LC-MS), and nuclear magnetic resonance (NMR) [15]. We utilized GC-MS to detect primary metabolites in the roots, stems, and leaves of *R. sachalinensis* and analyzed them to identify metabolites with significant differences.

Previous research has primarily focused on the extraction of *Rhodiola* compounds, antioxidant and anti-inflammatory activities, and radiation resistance. For example, Zhang, S.Q., and others conducted research on the extraction of active components from *Rhodiola* [16]. Choe, K.I., and colleagues studied the anti-inflammatory and antioxidant properties of *Rhodiola* [17], while Arora, R., and others investigated the radiation resistance of *Rhodiola* [18]. However, there has been relatively limited research on the genetic diversity of wild *R. sachalinensis* through morphological characteristics, with few recent reports in this area. Additionally, there is a lack of relational reports on the determination of primary metabolites in different plant parts. This study aims to analyze the genetic diversity relationship between different plant parts of *R. sachalinensis* from two different locations based on their morphological characteristics. It also aims to assess the genetic diversity of *R. sachalinensis* resources and reveal intrinsic connections between different plant parts using metabolomics. This research is of significant importance for the development of *R. sachalinensis* resources and genetic breeding efforts.

## 2. Materials and Methods

### 2.1. Materials and Reagents

The study area was the Pingding Mountain region of Dahailin, Heilongjiang Province, in the southeastern part of the Zhangguangcai Ridge, and Fenghuang Mountain, which is situated in the high mountain zone on the western slope of the Zhangguangcai Range. These mountains are situated in a high-altitude area with a distinct high-mountain climate, characterized as a temperate monsoon climate (Dwb). Winters are cold and dry, while summers are hot and receive more rainfall. In the winter, the climate is dominated by polar continental air masses, leading to extremely cold and dry conditions, while in the summer, the influence of tropical maritime air masses results in more concentrated precipitation, making the climate mild and humid. In this research, we selected high-altitude *R. sachalinensis* as the study subject in two locations: Pingding Mountain in Dahailin, Heilongjiang Province (128°23′06″ N, 44°26′31″ E) (Figures S1 and S2), and Fenghuang Mountain in Wuchang City, Heilongjiang Province (127°59′26″ N, 44°7′22″ E). Both locations have an elevation of 1550 m. Sampling and surveys were conducted in both areas in July 2023. During the survey, we employed a five-point sampling method, selecting 100 individual plants in each plot. We assessed multiple plant characteristics, encompassing plant height, leaf length, leaf width, and stem diameter. Plant height was determined by measuring the length of the primary stem (the tallest above-ground stem). Leaf length, leaf width, and leaf aspect ratio were measured on the largest leaves located in the upper one-third section of the primary stem. Stem thickness was gauged by measuring the diameter at the base of the primary stem. Measurements were conducted three times for each parameter, and the average value was calculated. The sampling followed the principle of protective sampling, where we selected six healthy, disease-free plants, consisting of two females, two males, and two hermaphrodite individuals. These plants were identified as *R. sachalinensis* by Professors Mu Liqiang and Zheng Baojiang, experts in plant taxonomy from Northeast Forestry University. All collected plants were carefully cleaned, frozen with liquid nitrogen, and stored at −80 °C until GC-MS analysis.

The chemicals and reagents used in the study included pyridine (analytical grade, Adamas), methanol (chromatography grade, CNW Shanghai, China), ribitol (purity ≥ 99%, SIGMA, St. Louis, MO, USA), methoxyamine hydrochloride (TCI), derivatization reagents (REGIS, Morton Grove, IL, USA), BSTFA (with 1% TMCS), and pure water.

### 2.2. Instruments and Equipment

First, gas chromatographic analysis was conducted using a high-performance gas chromatography system, an Agilent 7890A Gas Chromatograph (Agilent, Santa Clara, CA, USA), equipped with a temperature controller, a column thermostat, and a DB-5 chromatographic column. Centrifugation operations were performed using a Heraeus Fresco 17 High-Speed Centrifuge (Thermo Fisher Scientific, Waltham, MA, USA). Mass spectrometry analysis was carried out using a PEGASUS HT Mass Spectrometer (LECO, Joseph, Buchanan County, MI, USA), and sample weighing was performed with a BSA124S-CW Analytical Balance (Sartorius, Göttingen, Germany). Samples were stored in a Forma 900 series Ultra-Low-Temperature Freezer (Thermo Fisher Scientific, Waltham, MA, USA).

### 2.3. Sample Preparation and GS-MS Analysis

The extraction and GC-MS analysis of metabolic products from the leaf, stem, and root segments of *R. sachalinensis* followed the procedures described in previous relevant research [19]. The steps involved were as follows: Weigh 0.1 g of the collected *R. sachalinensis* samples from the root, stem, and leaves and place them in separate centrifuge tubes. Add 540 μL of methanol and 60 μL of the internal standard (L-2-chlorophenylalanine, 0.3 mg·mL^−1^) to each tube. Mix the contents and sonicate for 30 min. Add 600 μL of water and 300 μL of chloroform to each tube. Continue sonication for another 30 min. Centrifuge the tubes at 14,000 rpm for 10 min at 4 °C. Collect 700 μL of the supernatant from each tube. Add 200 μL of methoxyamine pyridine solution (15 mg·mL^−1^) to re-dissolve the supernatant and incubate at 37 °C for 90 min.

Add 200 μL of BSTFA (containing 1% TMCS) derivatization reagent and 40 μL of n-hexane to each tube. Vortex the samples for 2 min and then derivatize at 70 °C for 60 min.

Centrifuge the samples to extract the supernatant, which is ready for GC-MS analysis. For the GC-MS analysis, utilize an Agilent 7890A autosampler (Agilent, Santa Clara, CA, USA) combined with an Agilent 5975C GS-MS and a non-polar DB-5 capillary column (30 m × 250 μm ID, Thermo Fisher Scientific, Waltham, MA, USA) for separation and mass spectrometry detection. Use helium (He) as the carrier gas with a flow rate of 1.0 mL/min. Employ the following temperature program for column heating: 4 °C/min from 125 °C to 210 °C, 8 °C/min from 60 °C to 125 °C, and 10 ℃/min from 270 °C to 305 °C. Maintain the final temperature at 305 °C for 3 min. Set the injection port temperature to 260 °C, and the injection volume is 1 μL. Apply an ionization voltage of −70 V.

Perform mass scanning in the range of *m*/*z* 50–600 with a 5 min delay before starting data collection at a scanning speed of 20 spectra per second. This process allows for the analysis of the metabolites from the different plant parts of *R. sachalinensis* using GC-MS.

### 2.4. Data Processing

Plant genetic diversity survey data were input into Microsoft Excel from hard copy records, and subsequent data analysis was conducted using SPSS 26. On the other hand, the gas chromatography–mass spectrometry (GC-MS) data were transformed from raw machine data to CDF format using data analysis software (Agilent GC-MS 5975, Santa Clara, CA, USA, A.04.10) and uploaded to the XCMS platform for preprocessing, including peak identification, filtering, alignment, and more [20]. The data were then exported to Microsoft Excel and imported into SIMCA-P software (Umetrics, Malmö, Sweden, 14.1) for principal component analysis (PCA) and multivariate statistical analysis (OPLS-DA). The purpose of this data analysis was to compare different parts of *R. sachalinensis* using principal component analysis (PCA) and orthogonal partial least squares discriminant analysis (OPLS-DA) to determine the key compounds that play a significant role in explaining the differences in metabolites among these plant parts.

Orthogonal partial least squares discriminant analysis (OPLS-DA) was employed to compare the metabolic differences in different parts of *R. sachalinensis*, allowing for the identification of important key compounds that elucidate the extent of metabolic variations. Permutation tests were conducted to assess the validity of the OPLS-DA models regarding overfitting, and all models were subjected to 999 permutations to demonstrate the degree of fitting. Compounds obtained from the OPLS-DA model with Variable Influence on Projection (VIP) values greater than 1.0 and *p*-values less than 0.05 were considered potential biomarkers [21]. Advanced heatmap plots were generated using the OmicStudio tools at https://www.omicstudio.cn (accessed on 12 October 2023). Hierarchical clustering analysis of the heatmap was performed using the Metware cloud platform. Furthermore, GC-MS pathway analysis was conducted using MetaboAnalyst 5.0. Information on metabolic pathways was searched for in the Kyoto Encyclopedia of Genes and Genomes (KEGG) (http://www.genome.jp/kegg/, accessed on 20 October 2023), which is a valuable resource for searching for information on metabolic pathways and related information for metabolites.

## 3. Results

### 3.1. Analysis of Morphological Characteristics of R. sachalinensis

Data were collected from five fixed sample plots in the Pingding Mountain region of Dahailin, each containing 100 wild *R. sachalinensis*. The data included plant height, stem diameter, leaf length, and leaf width, and statistical analysis was conducted (Table 1). Additionally, a morphological comparison was made with data from 100 wild *R. sachalinensis* plants in the Fenghuang Mountain area (127°59′26″ N, 44°7′22″ E). These seedlings were collected before the flowering stage and could represent trait relationships within a population. The results indicate that in the Pingding Mountain region of Dahailin, the coefficient of variation for the five morphological traits of *R. sachalinensis* ranges from 19.80% to 37.20%. All these traits, including plant height, stem diameter, leaf length, and leaf width, have variation coefficients exceeding 20%. The average plant height (Height) is 144.20 mm, with a maximum of 271.00 mm, a minimum of 34.00 mm, and a coefficient of variation of 37.20%. Plant height exhibits significant variation. The average stem diameter (Stem diameter) is 2.33 mm, with the thickest stem being 4.74 mm, the thinnest stem being 0.96 mm, and a coefficient of variation of 31.7%. Stem diameter also exhibits relatively high variability. The average leaf length (Leaf length) is 25.05 mm, with a maximum of 48.48 mm, a minimum of 9.39 mm, and a coefficient of variation of 28.9%. Leaf length shows moderate variability. The average leaf width is 7.49 mm, with a maximum of 15.28 mm, a minimum of 3.78 mm, and a coefficient of variation of 26.6%. Leaf width exhibits moderate variability. The average length-to-width ratio of the leaf is 3.38, with a maximum ratio of 5.32, a minimum ratio of 1.37, and a coefficient of variation of 19.8%. The length-to-width ratio of the leaf is relatively stable and shows less variation.

In the Fenghuang Mountain area, during the same period, the variation degree of the five morphological traits of *R. sachalinensis* is different. The coefficients of variation for plant height, stem diameter, leaf length, and leaf width all exceed 20%, ranging from 11.40% to 25.65%. Specifically, the average plant height is 226.6 mm, with a maximum of 338 mm, a minimum of 104 mm, and a coefficient of variation of 25.66%. The average leaf length is 25.7 mm, with a maximum of 38.9 mm, a minimum of 15.7 mm, and a coefficient of variation of 21.91%. The average leaf width is 8.5 mm, with a maximum of 12.5 mm, a minimum of 5.5 mm, and a coefficient of variation of 21.00%. The average stem diameter is 2.9 mm, with a maximum of 4.7 mm, a minimum of 1.5 mm, and a coefficient of variation of 20.44%. The average length-to-width ratio of the leaf is 3.02, with a maximum ratio of 4.58 and a minimum ratio of 2.12. The kurtosis of the leaf length-to-width ratio is greater than 1, reaching 3.69 (Table 2).

A Kolmogorov–Smirnov (K-S) test was conducted on the morphological data of *R. sachalinensis* in the Pingding Mountain Dahailin region. The results indicate that leaf width does not follow a normal distribution (*p*-value of 0.04).

**Figure 1:**
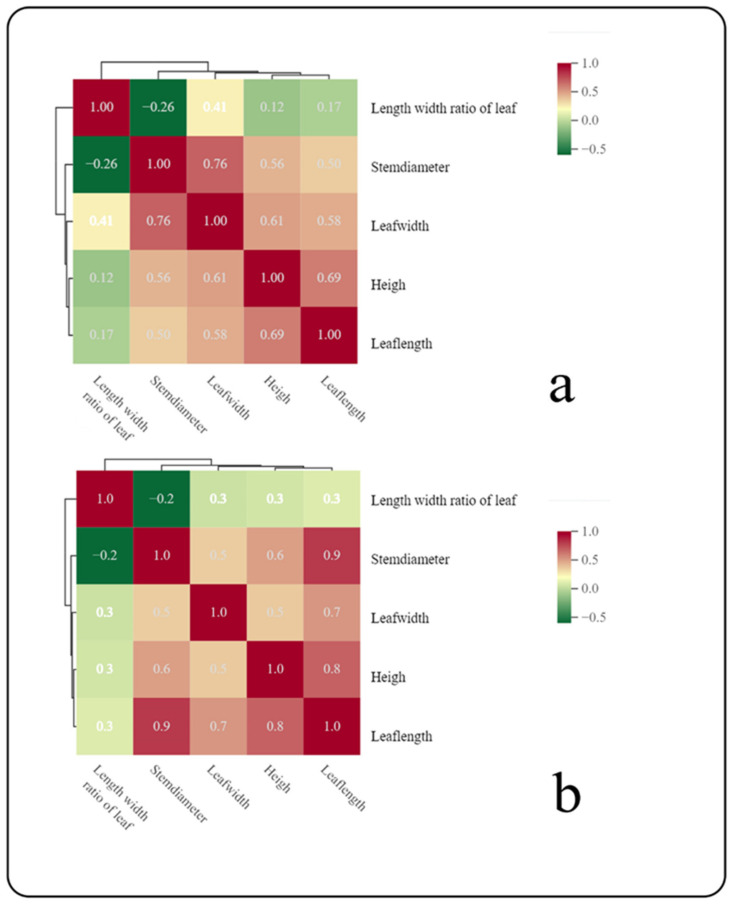
(**a**) Correlation clustering heatmap of morphological traits in *R. sachalinensis* from Pingding Mountain region of Dahailin. (**b**) Correlation clustering heatmap of morphological traits in *R. sachalinensis* from Fenghuang Mountain region of Wuchang City.

Under normal circumstances, various morphological traits in plants are interrelated, interact, and coordinate with each other throughout the plant’s growth process. Based on the coefficient of variation, and in order to further explore the relationships between different morphological traits, this study used Pearson correlation analysis in SPSS 26 and R software 3.5.3 to create a correlation clustering heatmap (Figure 1). From the clustering heatmap, it can be observed that during the same time period in the Pingding Mountain region, there is a highly significant correlation between leaf length and plant height. Additionally, there is a significant correlation between leaf width and leaf length and a significant correlation between leaf width and stem diameter. Plant height is moderately correlated with leaf width, leaf length, and stem diameter. Similarly, in the Fenghuang Mountain area, there is a strong correlation between leaf length and plant height, but an even stronger correlation is observed between leaf length and stem diameter.

Even though *R. sachalinensis* from the two locations were in the same growth stage and had similar elevations, there were still notable differences in morphological data. This suggests that even in cases where environmental differences are not significant, or under the influence of environmental conditions, there can be discernible morphological differences. This may be indicative of the plant’s sensitivity to environmental changes. To further investigate the significance of the morphological differences between *R. sachalinensis* from the two locations, the study continued with an analysis of 100 plants from both Pingding Mountain, Dahailin, and Fenghuang Mountain, Wuchang City. Single-factor analysis of variance (ANOVA) was employed (Table 3) to observe the significance of differences among different parts of *R. sachalinensis* in the two locations (The letters a, b, and c indicate that there is no significant difference between groups with the same letter, while there is a significant difference between groups with different letters). The results indicate that there are varying degrees of differences among different groups, suggesting that *R. sachalinensis* from the two locations exhibit differing levels of morphological variations. These variations are likely attributed to factors such as elevation or differences in soil composition.

### 3.2. PCA

**Figure 2:**
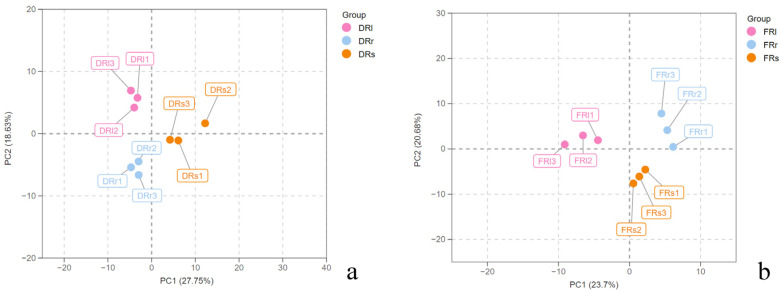
(**a**) The PCA score plot of *R. sachalinensis* roots (DRr), stems (DRs), and leaves (DRl) in Dahailin regions. (**b**) The PCA score plot of *R. sachalinensis* roots(FRr),stems(FRs), and leaves (FRl) in Fenghuangshan regions.

To distinguish the differences in the accumulation of metabolic products in the roots, stems, and leaves of *R. sachalinensis* from two different locations, a statistical approach was employed, starting with principal component analysis (PCA). PCA helps in better distinguishing differences among datasets by analyzing the internal structure of the data and explaining data variables. Initially, PCA was performed using SIMCA-P 14.1 software (Sartorius Stedim Data Analytics AB, Umea, Sweden). The obtained PCA model for Rhodiola sachalinensis from Dahailin showed a cumulative R2X of 0.991 and a cumulative Q2 of 0.782, while the model for *R. sachalinensis* from Fenghuang Mountain had a cumulative R2X of 0.913 and a cumulative Q2 of 0.568. These parameters, all exceeding 0.5, indicated the stability of the PCA models. The parameter values were within the confidence interval, falling within a reasonable range, making it possible to accurately differentiate the metabolic differences between the leaves and roots of *R. sachalinensis* for metabolomics experiments and metabolic difference analysis. Figure 2a shows that in the case of *R. sachalinensis* from Dahailin, the leaves (DRl) are distributed in the upper-left quadrant of the coordinate axes, the stems (DRs) are on the right side, and the roots (DRr) are in the lower-left quadrant. This distribution demonstrates that PCA effectively distinguishes the metabolic products of the leaves, stems, and roots of *R. sachalinensis*. As shown in Figure 2b, *R. sachalinensis* from Fenghuang Mountain exhibits variations in the distribution of metabolic products between different parts compared to Dahailin. The leaves (FRl) are on the left side of the coordinate axes, while the stems (FRs) and roots (FRr) are on the right side. While PCA can differentiate the principal components of each part, it has some limitations. Firstly, it can only differentiate principal components, which are sometimes ambiguous and do not explain the relationships between the contents of the principal components. Secondly, PCA is unsupervised, which can lead to the oversight of the specific roles of smaller components.

### 3.3. Analysis of OPLS-DA Model

**Figure 3:**
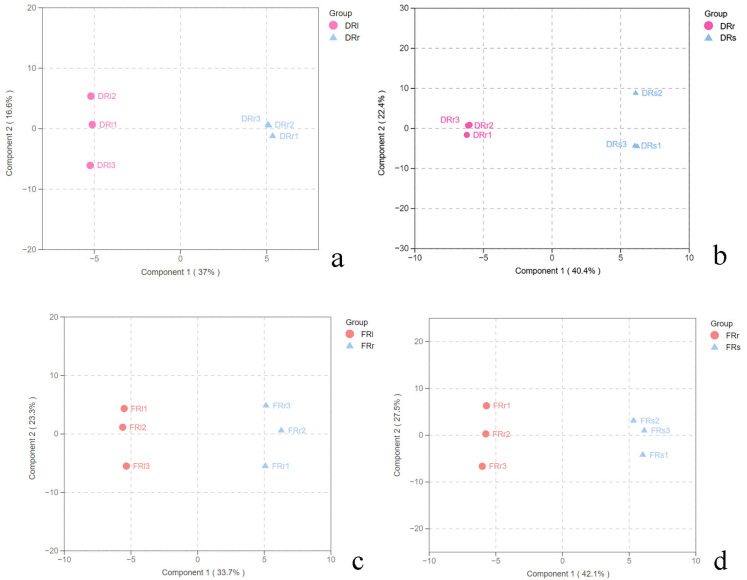
(**a**) The OPLS-DA scores of *R. sachalinensis* roots and leaves in Dahailinregions. (**b**) The OPLS-DA scores in *R. sachalinensis* roots and stems in Dahailin regions. (**c**) The OPLS-DA scores in *R. sachalinensis* roots and leaves in Fenghuangshan regions. (**d**) The OPLS-DA scores in *R. sachalinensis* roots and stems in Fenghuangshan regions.

The study indicates that the active component, salidroside, in Rhodiola rosea is primarily concentrated in the plant’s roots [22]. Therefore, further analysis was conducted using an orthogonal partial least squares discriminant analysis (OPLS-DA) model to examine the metabolic differences between the roots, leaves, and stems of Rhodiola rosea from the two different regions. The OPLS-DA model is a multivariate regression modeling method that can effectively remove data factors in the independent variable X that are unrelated to the categorical variable Y. It allows for the separate interpretation and analysis of orthogonal and non-orthogonal variables, providing a more accurate analysis of differences and correlations in metabolic products among different samples [23]. First, the data were preprocessed and subjected to UV transformation using SIMCA 14.1 software. Subsequently, modeling and analysis of the data were performed. The OPLS-DA score plots (Figure 3a,b) demonstrate a clear separation of primary metabolic products in the roots, stems, and leaves of Rhodiola rosea from Dahailin. This indicates that the OPLS-DA model can effectively distinguish the root samples from the other parts of Rhodiola rosea. Similarly, as seen in the score plots (Figure 3c,d), the primary metabolic product data from the roots and other two parts of Rhodiola rosea from Fenghuang Mountain also exhibit significant separation, indicating that the model can effectively differentiate these samples based on their metabolic profiles. The established OPLS-DA models include two main components. For Dahailin, the models differentiate between the root (DRr) and leaf (DRl) samples with R2Y = 0.995 and Q2 = 0.845 and between the root (DRr) and stem (DRs) samples with R2Y = 0.990 and Q2 = 0.878. For Fenghuang Mountain, the models distinguish between the root (FRr) and leaf (FRl) samples with R2Y = 0.995 and Q2 = 0.786 and between the root (FRr) and stem (FRs) samples with R2Y = 0.995 and Q2 = 0.768. All these model parameters exceed 0.5, meeting the expected criteria. This also indicates the metabolic differences among the different parts of Rhodiola rosea from the two regions.

Since the OPLS-DA model is a supervised classification model and overfitting is a normal occurrence in this context, the model is proficient at distinguishing samples from different parts. However, it may not perform optimally when used to predict new samples, which does not meet the desired requirements. To assess the reliability of the OPLS-DA model, permutation tests were conducted. The principle behind permutation tests involves shuffling the grouping order of each sample and then re-establishing a corresponding OPLS-DA model. This process allows for the calculation of a new Q2 value, and a relatively reliable model should have a Q2 value significantly higher than Q2 from random simulations [24].

#### Identification of Differential Metabolites

Differential metabolites were determined by analyzing the Variable Influence on Projection (VIP) values (VIP ≥ 1) and *t*-test results (*p* ≤ 0.05) in the context of the OPLS-DA model. In the Dahailin region, a total of 42 differential metabolites were identified between the roots and stems of *R. sachalinensis*, while 34 differential metabolites were identified between the roots and leaves. In the Fenghuang Mountain region, a total of 69 differential metabolites were identified between the roots and stems, and 50 differential metabolites were identified between the roots and leaves. These differential metabolites encompass a range of compounds, including sugars such as arabinofuranose, D-cellobiose, D-fructose, D-glucopyranoside glycosides, and arabinitol sugar alcohols, as well as amino acids like aspartic acid, L-serine, L-threonine, and L-valine. Carboxylic acids such as 2-butenedioic acid, caffeic acid, and citric acid were also among the differential metabolites, along with various quinones, esters, and saponin compounds.

**Figure 4:**
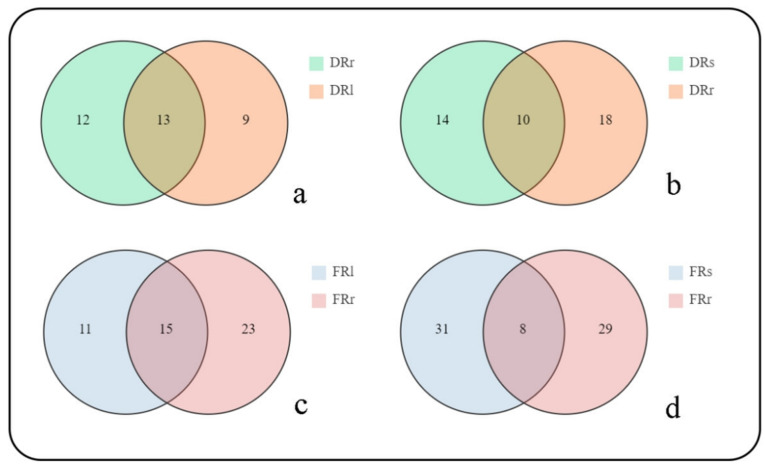
(**a**) Venn diagram of differential metabolites between roots and leaves of *R. sachalinensis* in the Dahailin region. (**b**) Venn diagram of differential metabolites between roots and stems of *R. sachalinensis* in the Dahailin region. (**c**) Venn diagram of differential metabolites between roots and leaves of *R. sachalinensis* in the Fenghuang Mountain region. (**d**) Venn diagram of differential metabolites between roots and stems of *R. sachalinensis* in the Fenghuang Mountain region.

Although the differential metabolites have been identified, their relationships among different plant parts are not entirely clear. Therefore, a Venn diagram was employed to provide a more intuitive understanding of the relationships between differential metabolites among various parts. In the Venn diagrams (Figure 4a,c), the numbers represent the counts of differential metabolites, and the overlapping sections indicate the shared differential metabolites. These shared metabolites may be involved in similar metabolic pathways. The Venn diagrams show that both regions exhibit significant differences in the overall count of differential metabolites between roots and leaves and between roots and stems (Figure 4b,d). While the Venn diagrams provide a preliminary overview of the quantity of differential metabolites, further research is needed to determine which compounds are responsible for these metabolic differences. Therefore, this study utilized hierarchical cluster analysis through heatmap visualization to more intuitively observe the relationships and changes in differential metabolites between the roots and other plant parts in different regions, aiming to identify the true underlying causes of these differences.

**Figure 5:**
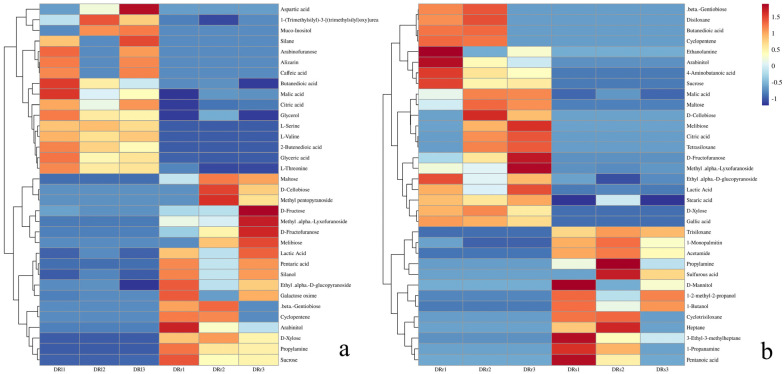
(**a**) Heatmap hierarchical clustering of differential metabolites between the roots and leaves of *R. sachalinensis* in the Dahailin area. (**b**) Heatmap hierarchical clustering of differential metabolites between the roots and stems of *R. sachalinensis* in the Dahailin area. In these heatmaps, red indicates high abundance, while low-abundance compounds are represented by blue (color scale key above the heatmap).

Heatmap hierarchical clustering analysis was performed to assess the relative changes in various differentially accumulated metabolites. After log transformation and normalization, the heatmap hierarchical clustering analysis categorized the differential metabolites into upregulated and downregulated groups. This analysis allows for a visual representation of the accumulation patterns in the roots, leaves, and stems of *Rhodiola sachalinensis* (Figure 5). From the results of the hierarchical clustering analysis, it is evident that there are significant differences in the differential metabolites identified in the two sample sets. In the leaves of Rhodiola sachalinensis, the accumulation of compounds including quinones such as alizarin and various sugars such as muco-inositol and arabinofuranose is significantly upregulated, indicating higher relative levels. On the other hand, the accumulation of sugars like maltose, melibiose, D-cellobiose, D-fructose, D-fructofuranose, D-xylose, galactose oxime, and sucrose, as well as glycosides like ethyl α-D-glucopyranoside and methyl α-lyxofuranoside, and acids like lactic acid and pentaric acid, and arabinitol is significantly upregulated in the roots of Rhodiola sachalinensis, showing higher relative levels. Similarly, when comparing stems to roots, the main upregulated compounds in the stems include siloxanes, mono-palmitin, acetamide, propylamine, D-mannitol, 1-2-methyl-2-propanol, 1-butanol, pentanoic acid, and sulfurous acid. For the remaining portions, the roots show significant upregulation of compounds such as maltose, sucrose, D-fructofuranose, D-xylose, butanedioic acid, citric acid, malic acid, ethanolamine, and 4-aminobutanoic acid. This suggests that sugars, amino acids, and fatty acid compounds are significantly upregulated in the roots compared to the other two parts, indicating that the roots are more active in sugar metabolism during primary metabolism. Heatmap hierarchical clustering analysis provides a clearer understanding of the changes in differential metabolites among the various plant parts and highlights the potential reasons behind the metabolic differences between these parts.

**Figure 6:**
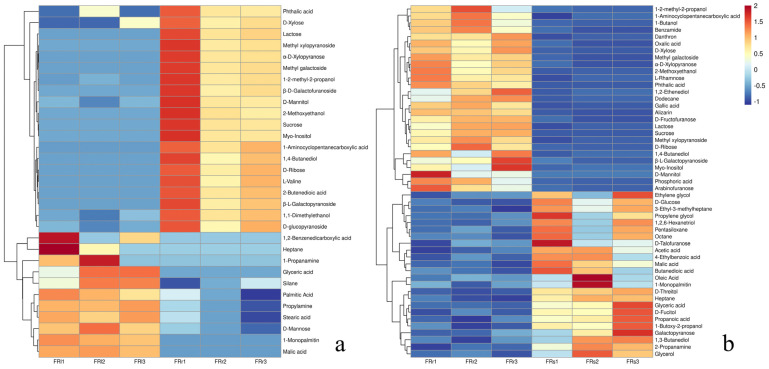
(**a**) Hierarchical clustering heatmap of differential metabolites between the root and leaf of *R. sachalinensis* in the Fenghuang Mountain region. (**b**) Hierarchical clustering heatmap of differential metabolites between the root and stem of *R. sachalinensis* in the Fenghuang Mountain region. In these heatmaps, red color represents high abundance, while blue color indicates low relative compound levels (color scale key above the heatmap).

In Figure 6, it can be observed that primary metabolites in the leaf part such as organic acids like 1,2-benzenedicarboxylic acid, glyceric acid, and malic acid; fatty acids like palmitic acid and stearic acid; and compounds like 1-propanamine and D-mannose are distinctly clustered together, showing significant upregulation compared to the primary metabolites in the root part. On the other hand, compounds such as 1-2-methyl-2-propanol, 1,4-butanediol, D-mannitol, and 2-methoxyethanol; alcohols; sugars like sucrose, D-ribose, and α-D-xylopyranose; and carboxylic acids like 2-butenedioic acid and phthalic acid, along with L-valine, form another cluster. These compounds are significantly upregulated in the root part compared to the leaf part. The hierarchical clustering heatmap makes it evident that there are substantial differences in metabolites between different plant parts, further indicating that different metabolic pathways are active in these distinct parts.

### 3.4. KEGG Metabolic Pathway Analysis

**Figure 7:**
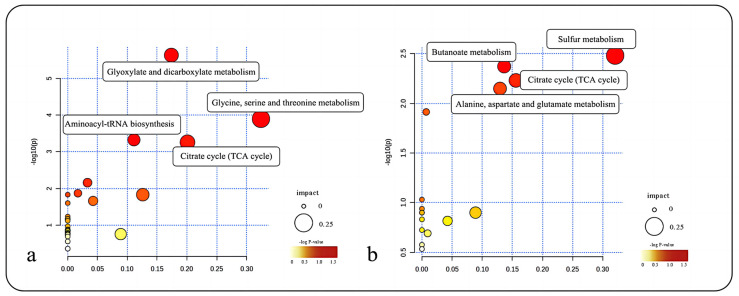
(**a**) KEGG metabolic pathway bubble chart for root and leaf of *R. sachalinensis* in Pingding Mountain region. (**b**) KEGG metabolic pathway bubble chart for root and stem of *R. sachalinensis* in Pingding.

To further analyze the metabolic pathways in different parts of *R. sachalinensis*, metabolites with differences from the GC-MS data were imported into MetaboAnalyst 5.0 for KEGG metabolic pathway analysis. It was found that the metabolic pathway differences between the roots and leaves of *R. sachalinensis* in the Dahailin region (Figure 7a) mainly included glyoxylate and dicarboxylate metabolism; glycine, serine, and threonine metabolism; aminoacyl-tRNA biosynthesis; and the citrate cycle (TCA cycle). The metabolic differences between the roots and stems (Figure 7b) included sulfur metabolism; the citrate cycle (TCA cycle); butanoate metabolism; and alanine, aspartate, and glutamate metabolism.

**Figure 8:**
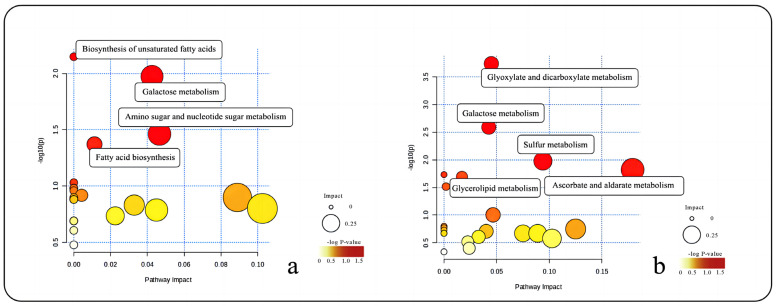
(**a**) KEGG metabolic pathway bubble chart for root and leaf of *R. sachalinensis* in Fenghuang Mountain region. (**b**) KEGG metabolic pathway bubble chart for root and stem of *R. sachalinensis* in Fenghuang Mountain region.

Similarly, KEGG metabolic pathway analysis for the roots and leaves of *R. sachalinensis* in the Fenghuang Mountain region revealed that the differences mainly centered around the biosynthesis of unsaturated fatty acids, galactose metabolism, amino sugar and nucleotide sugar metabolism, and fatty acid biosynthesis (Figure 8a). The differences between the roots and stems (Figure 8b) were mainly found in glyoxylate and dicarboxylate metabolism, galactose metabolism, sulfur metabolism, glycerolipid metabolism, and aldarate metabolism.

In a similar manner, KEGG metabolic pathway analysis was conducted for the *R. sachalinensis* roots and leaves from the Fenghuang Mountain region. The analysis revealed that the metabolic differences between the roots and leaves in this region (Figure 8a) were primarily concentrated in several significant metabolic pathways, including “Biosynthesis of unsaturated fatty acids”, “Galactose metabolism”, “Amino sugar and nucleotide sugar metabolism”, and “Fatty acid biosynthesis”. Conversely, the differences between the roots and stems (Figure 8b) were primarily associated with five metabolic pathways: “Glyoxylate and dicarboxylate metabolism”, “Galactose metabolism”, “Sulfur metabolism”, “Glycerolipid metabolism”, and “Aldarate Metabolism”.

## 4. Discussion

In previous research, morphological studies on wild *R. sachalinensis* have been relatively limited. However, morphological analysis remains a fundamental and direct approach in exploring plant genetic diversity [9,25]. Numerous scholars both domestically and internationally have utilized phenotypic traits to analyze the diversity of genetic resources in various plant species. For example, KJÆR ANDERS (2004) conducted research on the phenotypic morphology and genetic relationships of sago palm (Metroxylon sagu Rottb.) [10]. YP Du (2014) and colleagues conducted surveys and evaluations of resources in the lily genus [11], while FENG Li (2009) surveyed Chinese wild roses’ genetic resources [26]. Yong-He C (2008) and others conducted a morphological trait survey on populations of Siberian wild rye in Xinjiang [27]. Moreover, metabolic activities often vary significantly among different plant parts, and the same plant species in different geographical locations can exhibit varying metabolic differences [28]. Zhang, ZY et al. for instance, conducted a study on the differences in phenolic substances among different parts of Medicago sativa [29], and Yisimayili et al. described the metabolic characteristics of different parts of pomegranates [30]. Investigating the metabolic differences among different plant parts can provide a clearer understanding of the spatial metabolic characteristics of *R. sachalinensis* at the same point in time. These studies collectively highlight the importance of morphological analysis and metabolic profiling of different plant parts, as they contribute to a more comprehensive understanding of the plant genetic diversity and metabolic adaptations in response to varying environments.

Morphological data among different parts of *R. sachalinensis* populations in various sampling sites exhibit varying degrees of variability. Prior studies have suggested that higher coefficients of variation may indicate greater genetic diversity [31]. In the process of plant evolution, natural mutations often serve as a primary source of phenotypic variation [32]. Natural selection influences the existing genetic and phenotypic variation, as a plant’s long-term adaptation to different environmental conditions often passes on adaptive capabilities to its offspring through genetics. Consequently, over a longer period of evolution, this adaptation is inherited by the descendants [33]. Morphological analysis of primary traits not only allows the examination of external morphological features but also permits the investigation of how different traits are influenced by ecological behaviors, growth and development, and the specific environments in which they occur. Populations of wild *R. sachalinensis* from two distinct geographic locations displayed varying degrees of coefficients of variation among different plant parts. This indicates that both *R. sachalinensis* populations from these two locations possess rich genetic diversity. Through the investigation of wild plant resources and the observation of morphological traits, it becomes possible to gain a more intuitive understanding of the growth and development of *R. sachalinensis* resources in high-altitude areas of Northeast China, as well as the developmental status of wild plant communities.

Past research has shown that the genotype of plants can change plants’ external morphological characteristics in response to the environment, while genetic variation often affects the biosynthetic features of plants [34]. The metabolic differences between different plant parts provide valuable insights into the biosynthetic pathways of these plants. Through differential metabolites and KEGG metabolic pathways, it can be observed that the accumulation of carbohydrates, amino acids, and fatty acid compounds in the roots is significantly higher compared to the other two parts. Compounds like maltose, melibiose, D-fructose, and D-fructofuranose are essential metabolites that play a substantial role in plant development, growth, and morphogenesis [35]. Amino acids are often involved in intercellular transport and serve as valuable catalysts for carbohydrate synthesis [36]. Amino acid derivatives such as ethanolamine and 4-aminobutanoic acid are often directly proportional to carbohydrate metabolism [37], and this is prominently reflected in the metabolic profile of the root sections of wild *R. sachalinensis* in these two regions. Interestingly, changes in fatty acid composition are also predominantly observed in the roots. Enrichment in the fatty acid metabolic pathway has been linked to cold tolerance in plants in previous studies [38]. This indicates that the underground parts, particularly the roots, might produce more of these substances in response to low temperatures compared to the above-ground parts.

The tricarboxylic acid (TCA) cycle is not only the primary metabolic pathway for aerobic processes in biological systems, but it also serves as a crucial indicator of energy sources and growth and development. This process not only provides essential precursor materials for the biosynthesis of amino acids and nitrogen metabolism but also is linked to carbohydrate metabolism [39]. This study also indicates a positive correlation between the TCA cycle and carbohydrate and amino acid metabolism, implying that these metabolic pathways are often coordinated in the metabolic activity of *R. sachalinensis* roots. Sulfur metabolism suggests that the nutrients absorbed by the roots may serve as raw materials for the synthesis of sulfur-containing compounds in the roots, as well as many other functions related to plant growth and development [40]. In this research, we have uncovered an intriguing phenomenon: even in small-scale ecological changes, there are significant variations in the metabolism of *R. sachalinensis*. This highlights that even minor changes in habitat conditions can have a noticeable impact on a plant’s metabolic activities.

## 5. Conclusions

We utilized survey data of wild *R. sachalinensis* populations and GS-MS technology to uncover the genetic diversity of these resources in two different locations. Additionally, we explored the metabolic differences among various plant parts. Our findings reveal substantial metabolic variations between different parts of *R. sachalinensis*, including the tricarboxylic acid (TCA) cycle, carbohydrate metabolism, amino acid metabolism, and fatty acid metabolism. This suggests that different metabolic activities in various parts serve distinct functions. Simultaneously, it appears that the roots play a more significant role in substance synthesis and transport, showing more robust metabolic activities, which could explain the larger volume of *R. sachalinensis* roots compared to above-ground parts. 

## Figures and Tables

**Table 1:** Morphological analysis of *R. sachalinensis* in the Pingding Mountain, region of Dahailin.

	Height	Stem Diameter	Leaf Length	Leaf Width	Length–Width Ratio of Leaf
Mean (mm)	201.20	2.33	25.05	7.49	3.38
Standard Deviation (mm)	53.70	0.74	7.25	1.99	0.66
Minimum (mm)	104.00	0.96	9.39	3.78	1.37
Maximum (mm)	271.00	4.74	48.48	15.28	5.32
Kurtosis	−0.68	0.21	0.69	3.15	0.86
Skewness	0.08	0.55	0.53	1.30	−0.34
Coefficient of Variation (%)	37.2%	31.7%	28.9%	26.6%	19.8%
Asymp. Sig. (2-tailed)	0.70	0.66	0.39	0.04	0.84

**Table 2:** Morphological analysis of *R. sachalinensis* in the Fenghuang Mountain, region of Wuchang.

	Height	Stem Diameter	Leaf Length	Leaf Width	Length–Width Ratio of Leaf
Mean (mm)	226.6	2.9	25.7	8.5	3.02
Standard Deviation (mm)	58.1	0.6	5.6	1.8	0.34
Minimum (mm)	104	1.5	15.7	5.5	2.12
Maximum (mm)	338	4.7	38.9	12.5	4.58
Kurtosis	−0.86	−0.08	−0.72	−0.59	3.55
Skewness	−0.04	0.34	0.28	0.51	0.89
Coefficient of Variation (%)	25.66%	20.44%	21.91%	21.00%	11.40%
Asymp. Sig. (2-tailed)	0.18	0.00	0.06	0.06	0.20

**Table 3:** Comparison of the morphological differences of *R. sachalinensis* in the two regions.

Trait		Pingding Mountain, Dahailin	Fenghuang Mountain Wuchang
Height (mm)	Mean	201.20 ± 53.70 b	226.6 ± 58.1 a
	Coefficient of variation (%)	37.2%	25.66%
	Asymp. Sig. (2-tailed)	0.70	0.18
Stem diameter (mm)	Mean	2.33 ± 0.74 a	2.9 ± 0.6 b
	Coefficient of variation (%)	31.70%	20.44%
	Asymp. Sig. (2-tailed)	0.66	0.00
Leaf length (mm)	Mean	25.05 ± 7.25 c	25.7 ± 5.6 b
	Coefficient of variation (%)	28.90%	21.91%
	Asymp. Sig. (2-tailed)	0.39	0.06
Leaf width (mm)	Mean	7.49 ± 1.99 a	8.5 ± 1.8 b
	Coefficient of variation (%)	26.60%	21.00%
	Asymp. Sig. (2-tailed)	0.04	0.06
Length–width ratio of leaf	Mean	3.38 ± 0.66 a	3.02 ± 0.34 a
	Coefficient of variation (%)	19.80%	11.40%
	Asymp. Sig. (2-tailed)	0.84	0.20

## Data Availability

The original contributions presented in the study are included in the article/Supplementary Materials, further inquiries can be directed to the corresponding authors.

## Supplementary Materials

The following supporting information can be downloaded at https://www.mdpi.com/article/10.3390/plants13040467/s1, Figure S1: (A–D) shows the images of high-altitude *R. sachalinensis* species. In Pingding Mountain Region of Dahailin; Figure S2: Habitat of *R. sachalinensis* in Pingding Mountain Region of Dahailin. Figure S3: The growth of associated plants under the forest in PingDing Mountain Region of DaHaiLin.
