# Supplementary material for: Morphological Variation and Spatial Metabolic Variations in Rhodiola sachalinensis A.Bor. in Different Natural Distribution Areas
Source: Plants (Basel). 2024 Feb 6;13(4):467. doi: 10.3390/plants13040467 (PMC10892287; doi:10.3390/plants13040467)
Supplement: Supplementary file 1 [file plants-13-00467-s001.zip › plants-2799410-supplementary.pdf]

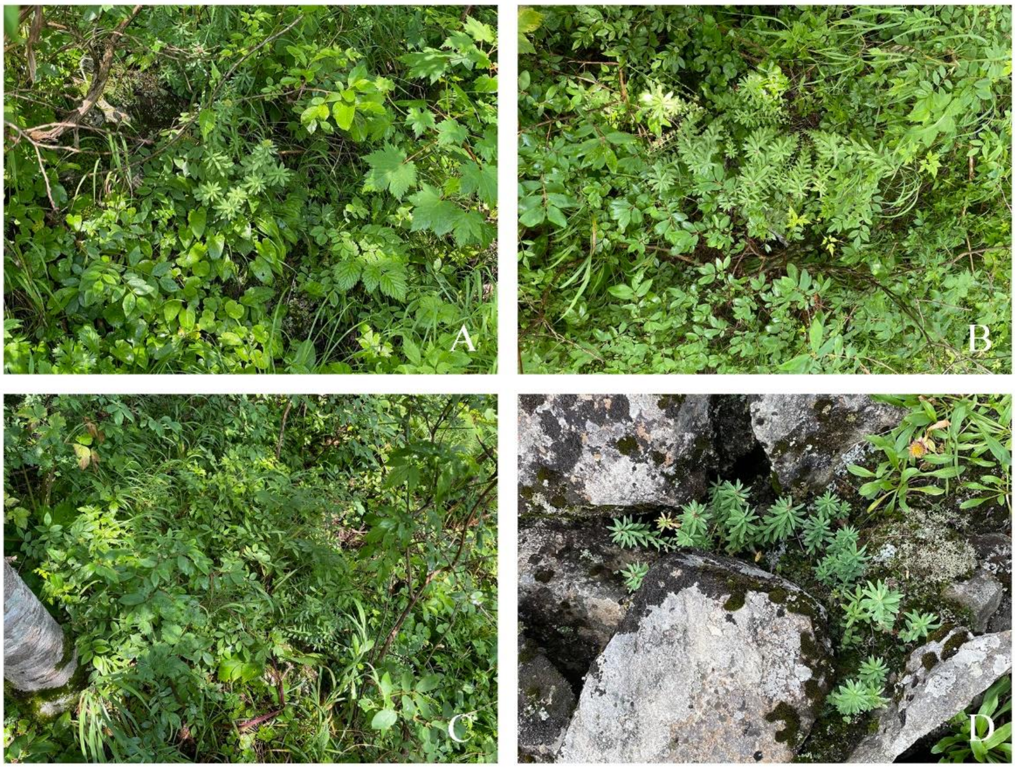

Figure S1. Figure A B C D shows the images of high-altitude *R. sachalinensis* species. In PingDing Mountain Region of DaHaiLin.

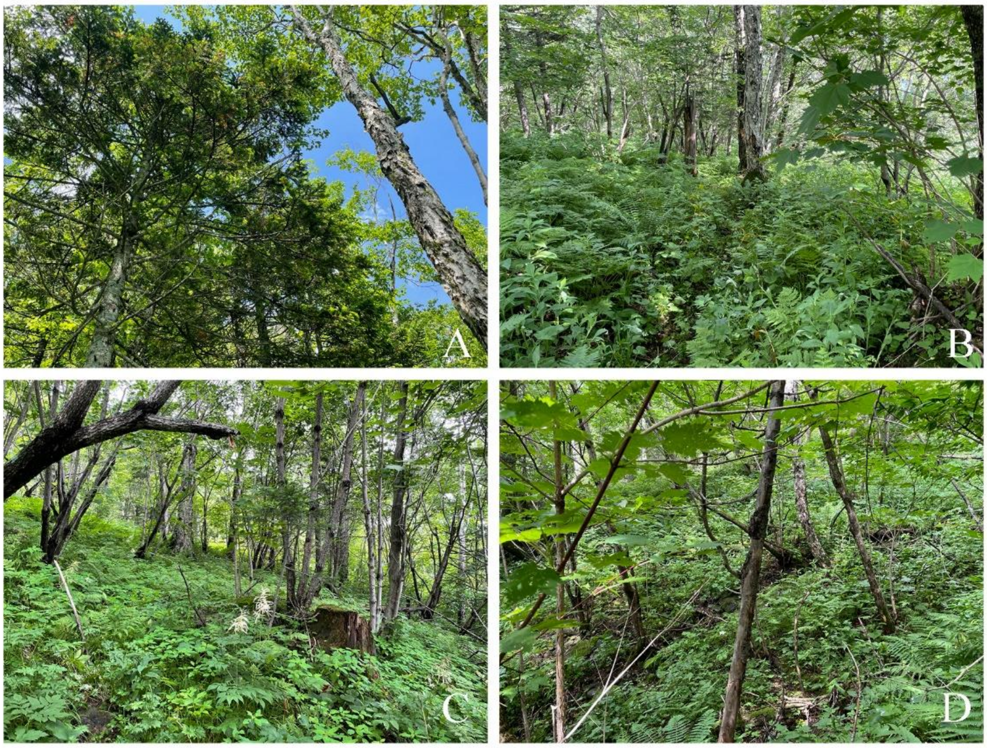

Figure S2. Habitat of *R. sachalinensis* in PingDing Mountain Region of DaHaiLin.

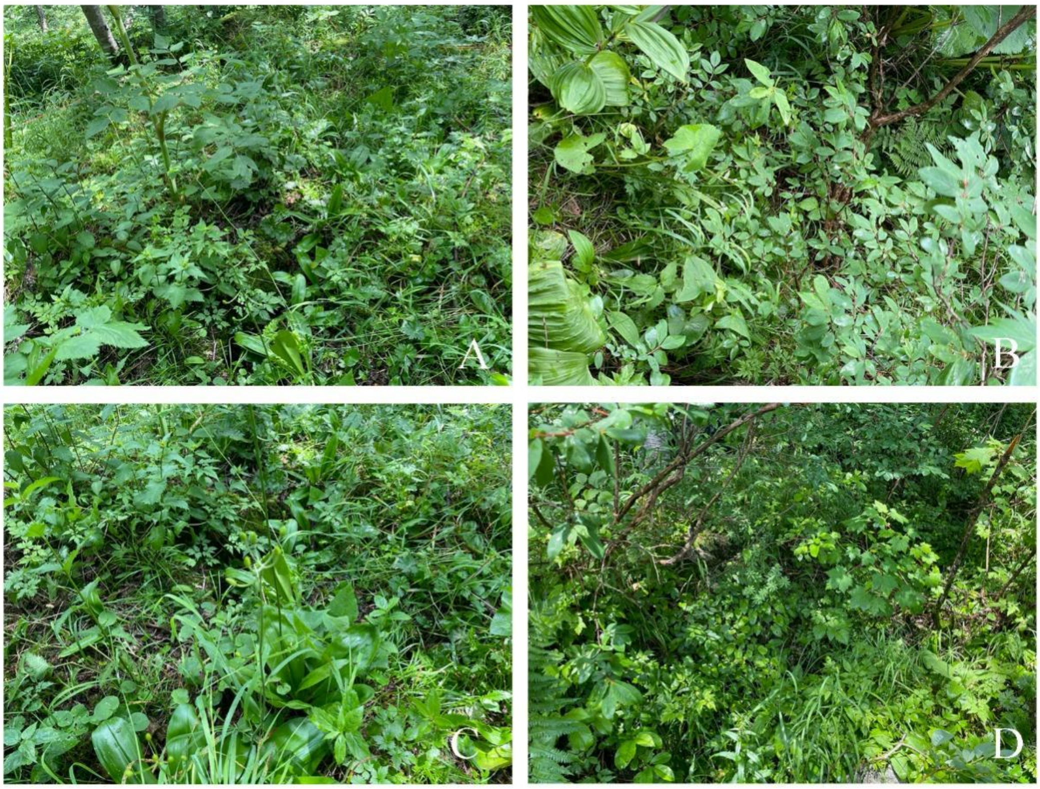

Figure S3. The growth of associated plants under the forest in PingDing MountainRegion of DaHaiLin.
